# Effects of Myostatin b Knockout on Offspring Body Length and Skeleton in Yellow Catfish (*Pelteobagrus fulvidraco*)

**DOI:** 10.3390/biology12101331

**Published:** 2023-10-12

**Authors:** Xincheng Zhang, Fang Wang, Mi Ou, Haiyang Liu, Qing Luo, Shuzhan Fei, Jian Zhao, Kunci Chen, Qingshun Zhao, Kaibin Li

**Affiliations:** 1 Key Laboratory of Tropical and Subtropical Fishery Resources Application and Cultivation, Ministry of Agriculture and Rural Affairs, Pearl River Fisheries Research Institute, Chinese Academy of Fishery Sciences, Guangzhou 510380, China; zhangxincheng2012@126.com (X.Z.);; 2 Model Animal Research Center, Nanjing University, 12 Xuefu Road, Pukou High-Tech Development Zone, Nanjing 210061, China; 3 Institute of Genome Editing, Nanjing YSY Biotech Company, No. 1 Amber Road, Nanjing 211812, China

**Keywords:** myostatin b, genome editing, *Pelteobagrus fulvidraco*, body length, skeleton, vertebral centrum

## Abstract

**Simple Summary:**

Myostatin is a negative regulator of muscle growth and development, and mutational inactivation of this gene increases muscle mass. Knockout of the *mstnb* gene in *Pelteobagrus fulvidraco* did not increase muscle mass, although our previous study of the *mstna* gene did. Instead, it reduced the body size and growth performance of *Pelteobagrus fulvidraco*. Through the present study, we found that the mutant *Pelteobagrus fulvidraco* had a reduction in the number of vertebrae, the length, and the intervertebral distance in *Pelteobagrus fulvidraco*, and these changes may be the underlying cause of the shorter body length in mutant *Pelteobagrus fulvidraco*. Histological comparison of the same sites in the *mstn* mutant and wild groups of *Pelteobagrus fulvidraco* also revealed that the number and density of osteocytes were greater in *mstnb* knockout *Pelteobagrus fulvidraco* than in wild-type *Pelteobagrus fulvidraco*.

**Abstract:**

Based on obtaining *mstnb* gene knockout in *Pelteobagrus fulvidraco*, a study on the effect of the *mstn* gene on skeletal morphology and growth was performed by comparing the number and length of the vertebrae of mutant and wild-type fish in a sibling group of *P. fulvidraco*, combined with the differences in cells at the level of vertebral skeletal tissue. It was found that *mstnb* gene knockdown resulted in a reduction in the number of vertebrae, the length, and the intervertebral distance in *P. fulvidraco*, and these changes may be the underlying cause of the shorter body length in mutant *P. fulvidraco*. Further, histological comparison of the same sites in the *mstn* mutant and wild groups of *P. fulvidraco* also revealed that the number and density of osteocytes were greater in *mstnb* knockout *P. fulvidraco* than in wild-type *P. fulvidraco*. Our results demonstrated that when using genome editing technology to breed new lines, the effects of knockout need to be analyzed comprehensively and may have some unexpected effects due to insufficient study of the function of certain genes.

## 1. Introduction

Myostatin (MSTN), a secreted hormone mainly produced by skeletal muscle, is a negative regulator of muscle development and growth [1,2,3]. Spontaneous mutations in the *mstn* gene, causing skeletal muscle mass to increase, are found in many mammals [4,5,6]. Through selective breeding, Belgian Blue cattle and Piedmontese cattle, carrying a mutant myostatin gene (*mstn*), have become superior domestic animal strains. As an essential gene for improving production efficiency, the myostatin gene is given more attention in modern animal breeding.

The continuous improvement of gene editing technology has broad applications in basic research, breeding, and biomedicine. Much research on the applications of gene editing for breeding target traits in aquatic animals has been reported. Various mutations in fishes, shrimp, and other aquatic species have been obtained in recent years. Kim et al. [7] found that a zebrafish model with a functional deficiency of the Down syndrome gene showed social impairments relevant to autism. *EcMIH* gene deletion shortened the metamorphosis time from mysis larva to postlarva of *Exopalaemon carinicauda* [8]. Nie et al. [9] found a crucial role for *runx2b* in intermuscular bone formation using CRISPR-Cas9. To obtain individuals with a high meat yield, *mstn* gene editing in many kinds of fish was conducted. Recently, genome editing technology induced a disruption to the fish *mstn* gene in yellow catfish [10,11,12], common carp [13], channel catfish [14], zebrafish [15], medaka [16], red sea bream [17], and olive flounder [18]. They all displayed a double muscling phenotype, whereas the increased muscle mass was varied.

Yellow catfish (*Pelteobagrus fulvidraco Richardson*) is an important freshwater product in Chinese aquaculture [19,20,21]. The aquaculture production of yellow catfish in China was 536,964 tons in 2022 [22]. To improve the low meat yield, we have reported knockouts of the *mstna* and *mstnb* genes in yellow catfish [10,11] and found that the deletion of *mstna* resulted in the double-muscle phenotype in yellow catfish [12].

This study found that the yellow catfish carrying genome-edited *mstnb* exhibited no significant effect on muscle mass. However, it showed a shorter body length and a higher HBL ratio (head length/body length). Some studies pointed out that MSTN regulates skeletal muscle growth and closely relates to bone metabolism in mammals [23,24]. There are few reports about the effect of *mstn* mutations in fish bone phenotype or bone metabolism. Only Kishimoto et al. [17] found a new phenotype of a short body length and small centra in *mstn* knockout red sea bream, which is not observed in mice and other teleost fish. A shorter body length would decrease fish meat; thus, we investigated (1) the alterations in growth and shape in *mstnb* mutant yellow catfish; (2) the bone changes of the vertebral column in *mstnb* mutant yellow catfish; and (3) the possible mechanism of MSTN contribution to bone formation.

Further research will compare *mstna* and *mstnb* gene mutants to determine their functions and confirm the phenotype of the double mutants in yellow catfish. This research on the influence of *mstn* gene mutations on body length and bone development could comprehensively evaluate its effect on improving the *Pelteobagrus fulvidraco* aquaculture industry.

## 2. Materials and Methods

### 2.1. Artificial Insemination and Breeding of the Heritable Myostatin b Gene (mstnb) Mutation in Yellow Catfish

When founder yellow catfish [11] carrying the mutated allele with an 8 bp deletion (nju22) reached maturity, they were mated with wild-type partners to obtain F1 offspring. Then, F1 offspring (*mstnb*^nju22/+^) carrying the mutation were raised to adulthood and mated to obtain F2 homozygous offspring.

Artificial insemination and breeding of yellow catfish were performed using the procedures described previously [12]. LHRH-A2 (Ningbo Sansheng Pharmaceutical Co., Ltd., Ningbo, China) and Domperidone Injection Solution (Ningbo Second Hormone Factory, Ningbo, China) were used to promote the maturity of the parental fish. The eggs were collected by repeatedly massaging the female yellow catfish’s abdomen. The collected eggs were mixed immediately with minced testes removed from a male individual in a 0.75% NaCl solution. Then, the fertilized eggs were dispensed on the meshes in lab aquariums with aerated water at a density of approximately 300–500 eggs per aquarium (0.15 m^3^). The hatching water temperature was maintained at 28 °C in the lab aquarium. The hatching larvae were raised similarly to growing zebrafish for further screening. At 3–4 weeks post-hatching, the juvenile yellow catfish were transferred to a large tank for raising (3.2 m × 1.6 m × 0.3 m).

### 2.2. Genotyping of Yellow Catfish Carrying Mutated mstnb Allele (nju22)

Founder yellow catfish potentially carrying the heritable targeted inactivation of myostatin gene (*mstnb*) were identified using the methods described previously [12] (Zhang et al. 2020). Multiplex PCR amplification was used to identify the genotype and the gender of the yellow catfish. A tiny piece of caudal fin derived from the founder or offspring was clipped to quickly make the genomic template by directly submerging it into YSY buffer (YSY, China) and incubating it at 65 °C for 30 min, at 95 °C for 10 min, and at 16 °C for 1 min. A total of 1 μL of the lysis solution was then used as a PCR template to amplify the mstn fragment.

The PCR reaction contained 5 μL of Multiplex PCR Master Mix (Applied Biosystems, USA), 2 μL of 10 μM forward primers (5′-AGCTCGACCAGTGAGTCAGTCAAAGCGTATCTCAGACC-3′ and 5′-CGACAGACAGTAAGGTCTCTGAGAGATGAAGAAGGGGGACAAG-3′) and reverse primers (5′-TCTTTCCCTTGGCTGTCA-3′ and 5′-TTTCGGAACAGAGGGAGTGG-3′), and 20 μM fluorescence labelled probes (5′VIC-CAGGAACTCAGTGTGACACTC-3′, green fluorescence; and 5′-NEDCGACAGACAGTAAGGTCTCTG-3′, yellow fluorescence). The amplification conditions were 94 °C for 5 min; 26 cycles at 94 °C for 30 s, 56 °C for 40 s, and 72 °C for 40 s; 8 cycles at 94 °C for 30 s, 53 °C for 40 s, and 72 °C for 30 s; 72 °C for 2 min; and 4 °C for 5 min. The fragments were separated by capillary electrophoresis in an ABI 3031 Gene Sequencer and Gene-mapper V4.1 was used to calculate the size of each fragment.

Three different genotypes of *mstnb* in the F2 offspring were identified: a single blue peak (PCR fragment size was 347 bp) denoted a wildtype genotype; double blue peaks (PCR fragment sizes of 339 bp and 347 bp) indicate a heterozygote; and a single blue peak (PCR fragment size of 339 bp) represented the *mstnb* knockout homozygote. Any samples with unexpected results were reamplified and re-run for confirmation.

### 2.3. Morphological and Growth Analysis of Yellow Catfish

To observe the morphological phenotype of *mstnb* null yellow catfish, 600 offspring from *mstnb*^nju22/+^ yellow catfish were raised together in the lab aquarium (3.2 m × 1.6 m × 0.3 m) under the abovementioned conditions. The culture temperature was maintained at 24–28 °C, and special feed for *P. fulvidraco* was fed to them twice daily. At approximately 60 dpf (days post-fertilization), the PIT chip was implanted in the peritoneal cavity, and genotyping was performed by clipping the fins using the method described above. The morphological differences were observed and recorded every month. Morphological data were measured randomly in 300 experimental fish at a time. Body weight (the weight of fish without water), body length (the length from the tip of the snout to the caudal end of the tail fin), body height (the vertical distance at the top of a fish), body width (the maximum distance from the left to the right side of the fish body), and head length (the length from the tip of snout to the end of gill cover) of yellow catfish were measured at 90, 180, 270, and 360 dpf. Experimental data were normalized using the HBL ratio (head length/body length) to compare the differences in the length of the trunk between wild-type and *mstnb* null yellow catfish.

### 2.4. Skeletal Staining and Vertebral Counts

Wild-type and *mstnb* knockout yellow catfish at 90 (male, *n* = 30) and 360 (male, *n =* 31) dpf were used for vertebral counts. All individuals were euthanized using an overdose of the anesthetic MS-222 (Sigma Aldrich, Saint Louis, MO, USA).

Skeletal examination of juvenile specimens (5–10 cm) was performed by making the skeletal specimens transparent and staining with them Alizarin red (Aladdin, Shanghai, China) and Alcian blue (Aladdin, Shanghai, China) to identify bone and cartilage, using the methods described in references [25,26,27,28]. They were prepared by removing the skin, gills, and viscera. The skeleton of a 360 dpf individual was obtained directly by boiling and removing the muscle.

The specimens were dehydrated and fixed in 95% ethanol for 2–3 days, and then they were transferred into acetone for fat removal. After removing the acetone with water or 70% ethanol, Alcian blue and Alizarin red staining solutions were added to the specimens for one or two days at room temperature. For transparency and to allow the visualization of the stained skeletal elements, stained fish were immersed in a 0.5% potassium hydroxide (KOH) solution to hydrolyze soft tissues. After cleaning in 0.5% KOH, the fish were gradually transferred to 100% glycerin. Stained bone specimens were stored in 100% glycerin with a small grain of thymol as a preservative.

Vertebral counts were performed with the naked eye or a stereomicroscope (SDPTOP SZ). Counts and vertebral regionalization were performed according to the references [29,30]. The total vertebral number (Vt) included the vertebrae of the Weberian apparatus, precaudal vertebrae, and caudal vertebrae. The vertebral numbers in the three regions of the axial skeleton were also counted: the Weberian apparatus (the first six precaudal vertebrae are regionalized as the Weberian vertebrae in *Bagridae*, and the sixth Weberian vertebra is associated with the first rib), precaudal vertebrae (vertebrae from the seventh to the first anal fin pterygophore without Weberian vertebrae), and caudal vertebrae (vertebrae from the first anal fin pterygophore to vertebrae of the caudal complex).

### 2.5. Histological Analysis on the Vertebral Column of Yellow Catfish

**Figure 1:**
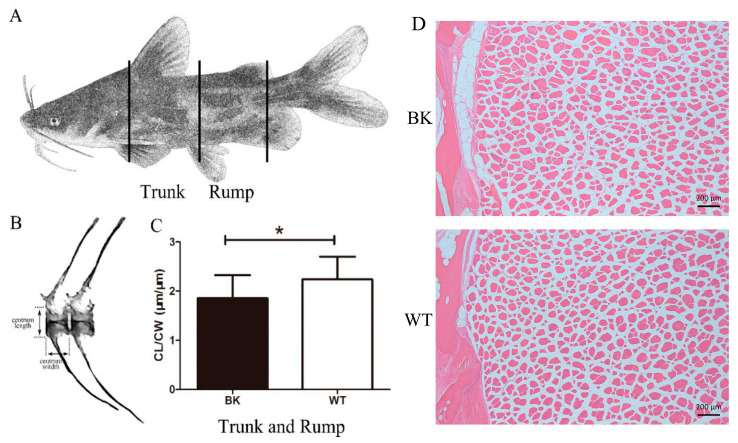
Schematic shows the slice positions and centrum parameter analysis at a yellow catfish fish. (**A**) Schematic showing the transverse and longitudinal slice positions (black lines) at a yellow catfish fish. (**B**) Schematic showing the centrum length and width at trunk and rump at 180 dpf. (**C**) Histograms showing the ratio of CL/CW (centrum length/centrum width) in the yellow catfish centrums at the trunk and rump. (**D**) Muscle sections from the same locations in the mutant and wild type showed no significant differences. Data are shown as mean ± SD (*n =* 3). * *p* < 0.05.

Wild-type and *mstnb* knockout yellow catfish juvenile (male, *n =* 3 individuals) at 90 dpf were selected for histological analysis of the vertebral column. The fish were sectioned transversely in three positions (dorsal fin, ventral fin, and adipose fin) and longitudinally in the parts (precaudal vertebrae and caudal vertebrae) of the body (Figure 1A). The fish were subdivided into tissue blocks, and fixed in Bouin’s solution. The vertebrae were decalcified with EDTA-2Na for thin sectioning and embedded in paraffin. Sagittal sections were cut 7 μm thick with a rotation microtome using tungsten carbide knives. The sections, stained with hematoxylin and eosin, were examined with a light microscope.

### 2.6. Quantitative Analysis of Centrum Variations

To quantitatively analyze centrum variations in the histological sections of yellow catfish, we used the method previously reported in red sea bream and medaka research [31,32]. The length and width of the centra were measured in longitudinal sections of the trunk and rump part (Figure 1B). The ratio of centrum length and centrum width (CL/CW) were normalized to analyze the differences between *mstnb* null yellow catfish and their wild-type siblings (Figure 1C).

The cross-sections of the trunk and rump were selected to analyze differences. The same areas of the centra were chosen from *mstnb* knockout yellow catfish and their wild-type siblings to count the osteocyte numbers. Cellularity was calculated as the number of osteocytes per cross-sectional muscle area. The bone area was measured from captured digital images using the software ZEN 2 (Carl Zeiss Microscopy GmbH, Oberkochen, Germany, 2011). The number and the size of muscle fibers were calculated by the ImageJ program (National Institution of Health, Bethesda, MD, USA).

### 2.7. Statistical Analysis

Statistical analysis of the data was performed using GraphPad Prism 8 (GraphPad Software Inc., Boston, MA, USA). Data were presented as mean ± standard deviation (SD). Datasets were examined for the normality and homogeneity of the variance test using the D’Agostino and Pearson test (K2) and F-test, respectively. For variables with a normal distribution, the independent samples *t*-test (unpaired two-tailed *p*-value) was utilized to compare two groups. The Mann–Whitney test was used to compare abnormal distributional variables between two groups. Owing to the number of statistical tests we performed, a Bonferroni correction for multiple testing [33] was applied. Three independent growth outcomes were tested: body weight, full length, body length, body height, body width. Therefore, the significance level *p* = 0.05 was divided by five, which provides a significance level corrected for multiple testing: *p* = 0.01.

## 3. Results

### 3.1. Yellow Catfish Carrying Mutated mstnb Have Normal Reproductive Capacity

**Figure 2:**
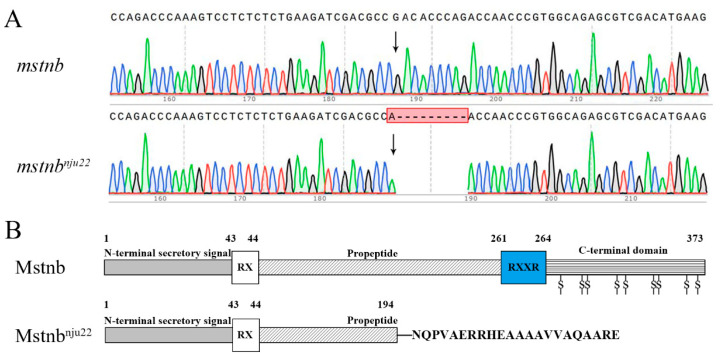
*mstnb* knockout yellow catfish were generated using TALENs. (**A**) Sequencing chromatogram showing the different mutated *mstnb* alleles between wild-type and *mstnb* knockout yellow catfish. Arrowheads indicate the mutated mstnb alleles were changed from the base. The red box shows the differences in partial *mstnb* DNA sequences and “-” shows the deletion of nucleotides. (**B**) Schematic showing mstnb proteins would be expressed in the wild-type and mutated yellow catfish. Mstnb: Wild-type mstnb protein contains a terminal secretory signal sequence, a propeptide domain, and a bioactive domain (C-terminal domain). RX is a cleavage site for proteolytic enzymes to remove the signal sequence and RXXR is a proteolytic processing site (RSSR) to produce the bioactive form of mstnb. The number shows the position of the amino acid residue. mstnb^nju22^: A truncated protein presumptively encoded in *mstnb*^nju22^ yellow catfish contains only the 194 amino acid residues of mstnb plus 22 new amino acid residues.

Previously, we generated six targeted disruptions (*mstnb*^nju20−25^) of *mstnb* in yellow catfish using Transcription Activator-Like Effector Nucleases (TALENs) [11]. Mutant *mstnb*^nju22^ yellow catfish carry a mutated allele with a nucleotide insertion (A) replacing 9 bp (GACACCCAG, nt978–986) in Exon 2 of *mstnb*. This mutant *mstnb^nju22^* allele was predicted to encode a truncated protein with only the 1st–192nd amino acid residues and an altered fragment (NQPVAERRHEAAAAVVAQAARE) due to a reading frame shift (Figure 2).

Artificial propagation of male *mstnb*^nju22/+^ and female *mstnb*^nju22/+^ yellow catfish was performed as described above. The fertilized eggs produced by *mstnb*^nju22/+^ (male) × *mstnb*^nju22/+^ (female) could develop normally to sexual maturity (F_2_), and the ratios of *mstnb*^+/+^, *mstnb*^nju22/+^, and *mstnb*^nju22/nju22^ were approximately 25%, 50%, and 25%, respectively. Both *mstnb*^nju22/nju22^ and *mstnb*^nju22/+^ yellow catfish were fertile and their offspring showed normal development. These results demonstrated that the *mstnb* gene is not vital for yellow catfish’s survival and reproduction.

### 3.2. Yellow Catfish Carrying mstnb Null Alleles Display Shorter Body Length

To verify whether the *mstnb* knockout affects growth, we recorded the growth characteristics of the F2 yellow catfish produced by *mstnb^nju22/+^* (male) × *mstnb^nju22/+^* (female) at 90, 180, 270, and 360 dpf (Table 1).

**Figure 3:**
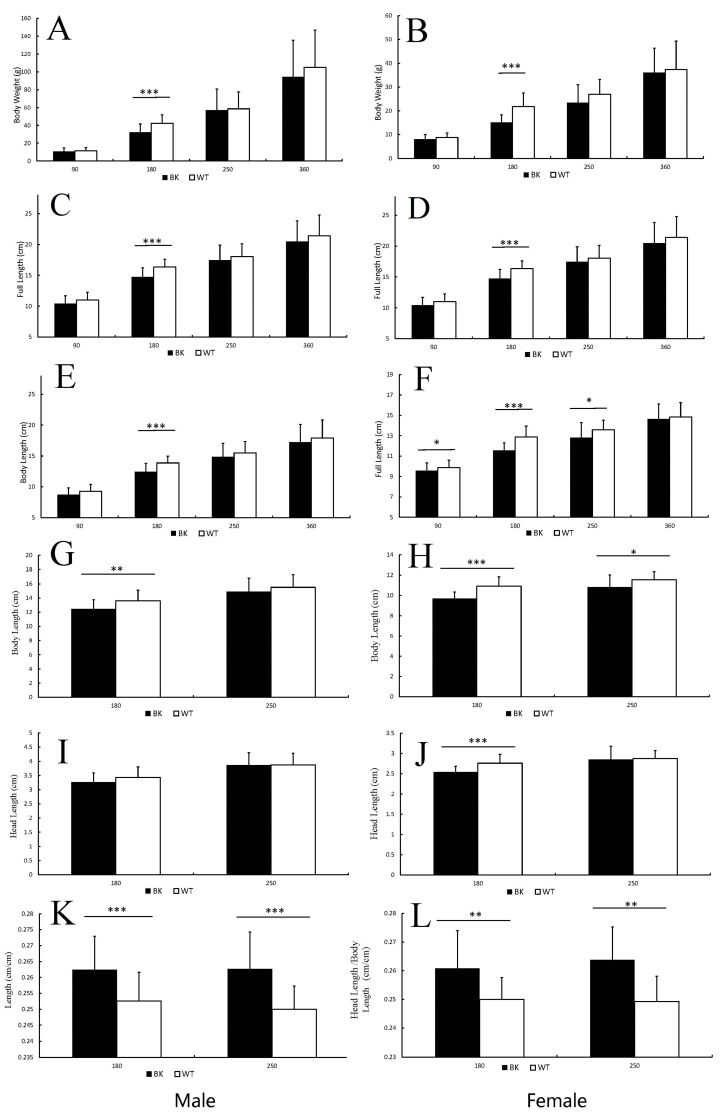
The morphological alterations in *mstnb* null yellow catfish compared with their wild-type siblings at 90, 180, 250, and 360 dpf. (**A**–**F**) *mstnb* knockout (BK) yellow catfish showed lower growth characteristics than the wild-type (WT). The full length (**C**,**D**) and body length (**E**,**F**) of BK yellow catfish were all shorter than the WT. (**G**–**J**) The head length and body length of the BK yellow catfish were further measured to confirm the position change in the BK at 180 and 250 dpf. The HBL ratios (head length/body length) showed BK yellow catfish has shorter trunk than their WT siblings (**K**,**L**). Data are shown as mean ± SD. BK: *mstnb*^nju22/nju22^; WT: *mstnb*^+/+^. * *p* < 0.05, ** *p* < 0.01, *** *p* < 0.001.

At 180 dpf, the body weight, full length, body length, body height, and body width of WT yellow catfish have significant differences with their *mstnb* KO siblings in males and females. Only the full length of WT yellow catfish has significant differences with their *mstnb* KO siblings in females at 90 and 250 dpf (Figure 3). At other stages of time, all growth parameters of WT yellow catfish were more significant than their *mstnb* KO siblings in male and female. However, statistical analysis revealed that there was no significant difference.

To confirm the body type alterations in the BK, the HBL ratio (head length/body length) of both male and female yellow catfish were measured at 180 and 250 dpf. The results showed that the HBL ratios of BK yellow catfish were 1.04 to 1.06-fold higher than their WT siblings (Figure 3K,L). At 180 dpf, the average head length of WT male yellow catfish was 5.21% longer than their male *mstnb* KO siblings (Figure 3I). The average head length of WT female yellow catfish was 8.67% longer than their female *mstnb* KO siblings (Figure 3J). At 250 dpf, the average head lengths of WT male and female yellow catfish were similar to their *mstnb* KO siblings (Figure 3J).

### 3.3. More Vertebral Centra in Wild-Type Yellow Catfish Than mstnb Knockout Yellow Catfish

**Figure 4:**
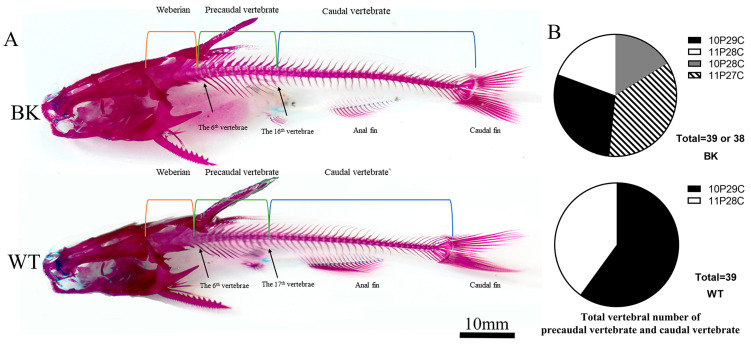
Skeletal staining and the percentages of the precaudal vertebrae and caudal vertebrae. (**A**) Skeletal staining of yellow catfish with Alcian blue and Alizarin red. Alcian blue and Alizarin red were used to identify cartilage and bone, respectively. The first six centrums formed the Weberian vertebrae, and the precaudal vertebrae were formed by the 7th–17th centrums in WT. (**B**) The pie chart with the percentages of precaudal vertebrae and caudal vertebrae in total vertebrae without Weberian vertebra. The 10P29C showed 10 precaudal vertebrae and 29 caudal vertebrae. P: Precaudal vertebrae; C: Caudal vertebrae. BK: *mstnb^nju22/nju22^*; WT: *mstnb^+/+^*.

Skeletal examination was performed using Alcian blue and Alizarin red staining to identify the bones and cartilage. The total vertebral number (Vt) of the wild-type and *mstnb* knockout yellow catfish showed significant differences. There were 45 vertebral centra in WT yellow catfish, whereas there were 44 or 45 centra (51.61%, 48.39%, respectively) in their BK siblings (Figure 4). There were six vertebrae in the Weberian complex in both genotypes, but the precaudal vertebrae or caudal vertebrae were missing a centrum in the BK. According to the classification standard of the precaudal vertebrae and caudal vertebrae above, the 17th centrum was a precaudal vertebrae (P) or caudal vertebrae (C) in different individuals. The proportion of WT yellow fish (*n =* 18) containing 10 precaudal vertebrae and 29 caudal vertebrae (P10C29) was 60.0%, while the proportion with P11C28 (*n =* 12) was 40.0%. In BK yellow fish (*n =* 31), there were four different vertebra compositions consisting of P10C28 (*n =* 5; 16.13%), P11C27 (*n =* 11; 35.48%), P10C29 (*n =* 9; 29.03%), and P11C28 (*n =* 6; 19.36%).

### 3.4. Centrum in Wild-Type Displayed Longer Length Than mstnb Knockout Yellow Catfish

**Figure 5:**
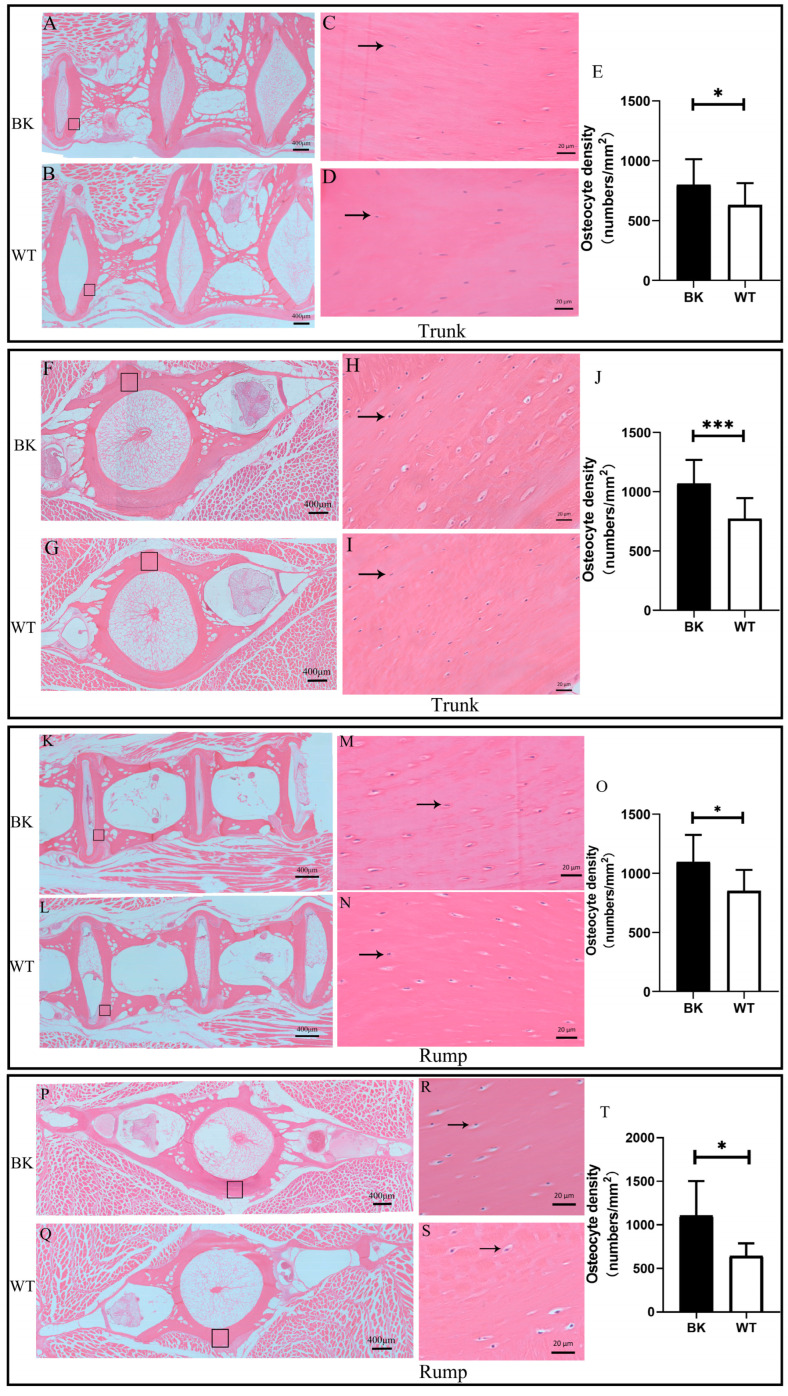
Histological analysis showed the centrum differences of osteocyte cellularity at the trunk and rump. (**A**–**D**,**F**–**I**) Transverse histological sections (hematoxylin and eosin staining) showed the number difference of osteocytes between wild-type and *mstnb* null yellow catfish at the trunk (**A**,**B**) and rump (**F**,**G**). The right pictures (**C**,**D**,**H**,**I**) were the amplified region of the centrum outlined by a black rectangular box of the left lines. (**K**–**N**,**P**–**S**) Longitudinal histological sections (hematoxylin and eosin staining) showed the number difference of osteocytes between wild-type and *mstnb* null yellow catfish in the trunk (**K**,**L**) and rump (**P**,**Q**). The right pictures (**M**,**N**,**R**,**S**) were the amplified region of the centrum outlined by the black rectangular box of the left lines. (**E**,**J**,**O**,**T**) Histograms showed the osteocyte cellularity in the transverse and longitudinal sections of yellow catfish centrums at the trunk and rump, respectively. The black histogram denotes the osteocytes of *mstnb* null yellow catfish the and white histogram denotes one of their wild-type siblings. Data are shown as mean ± SD (*n =* 3). Black arrows represent the osteocyte position in the centrums. * *p* < 0.05, *** *p* < 0.001.

To elucidate the effect of *mstnb* deficiency on the body length of yellow catfish, we performed longitudinal sections of the trunk and rump to compare the histological features in centrum length of *mstnb* knockout yellow catfish with their wild-type siblings (Figure 5A,B,F,G). A higher proportion of centrum length to width also showed that the wild-type centrum was significantly larger than that of *mstnb* knockout yellow catfish (Figure 1C).

### 3.5. mstnb Knockout Yellow Catfish Have Increased Osteocyte Number in Centrum

To further elucidate the effect of *mstnb* deficiency on the skeleton, we analyzed the bone cells in the centra at the trunk and rump. The transverse sections of centra at the trunk and rump both showed that there was a significant difference in osteocytes between *mstnb* knockout yellow catfish and their wild-type siblings.

In terms of osteocyte number, cellularity (osteocyte density) was used for a quantitative comparison between the *mstnb* null yellow catfish and their wild-type siblings. Lower values of cellularity mean that there are fewer osteocytes in a cross-section. As shown in Figure 5, the cellularity of osteocytes at the trunk and rump of *mstnb* null yellow catfish were higher than those of their wild-type siblings (Figure 5E,K). Overall, the results suggested that *mstnb* null yellow catfish had more osteocytes in their trunk and rump centra.

## 4. Discussion

*MSTN* is known to negatively regulate muscle development, and when it is deactivated, it induces an increase in muscle mass [34,35]. Since meat yield is an essential production indicator for fish, extensive efforts have been made to develop efficient methods to block *MSTN* expression and produce fish with increased muscle mass [36,37,38]. In our previous study, we knocked out *mstna* and *mstnb* genes in yellow catfish separately [10,11], and found that yellow catfish carrying knockout *mstna* displayed the double-muscling phenotype [12].

To assess the effects of *mstna* or *mstnb* genes on growth and muscle phenotype, we conducted a comparative experiment to evaluate the growth performance of yellow catfish carrying *mstna* or *mstnb* knockout (AK or BK). The *mstna* knockout yellow catfish had an increased muscle fiber number but decreased muscle fiber size in the skeletal muscle, whereas myostatin b null yellow catfish showed declining growth parameters and no increase in muscle mass compared with WT and *mstna* null yellow catfish [12]. The body length and HBL ratio suggested that the trunk of *mstnb* mutants was shorter than that of wild types. Furthermore, since the AK, BK, and WT yellow catfish were generated by the founders (*mstna*^+/−^*mstnb*^+/−^), there were few genetic differences between them as sibling groups. This phenomenon of body shape changes in the *mstnb* null yellow catfish appears to be caused by myostatin disruption.

The shortened trunk in myostatin b null yellow catfish was comparable to the red sea bream [17,36,37] and olive flounder [18] myostatin b mutants. Kishimoto et al. [17] found that *mstn* deficiency resulted in a slightly increased body width and height and shortened body length in red sea bream. Olive flounders carrying *mstn* null alleles were also slightly shorter and weighed more than the WT, but no significant differences were observed [18]. Considering that *P. olivaceus* continues to grow over five years, the authors suggested that the growth performance of the mutants should be elucidated with a larger sample size over an extended period of time.

It is worth noting that some reports about genome editing of *mstn* in other teleosts showed inconsistent results on body shape. The length of myostatin gene-edited common carp [13] and channel catfish [14] was significantly greater than wild types. The phenotypes of medaka carrying *mstn* null alleles showed an increased body weight, longer body length, and wider body width than their wild-type siblings [16,39]. Interestingly, the body lengths of *mstnb* gene knockdown zebrafish (with vector-based RNA interference) or *mstnb*-deficient zebrafish (with the TALENs technique) were both not significantly different from that of wild-type fish [40,41]. The body length of male zebrafish carrying *mstna* (similar to the *mstnb* gene in other fish) null allele increased slightly compared to the wild type at four months old. In contrast, male zebrafish carrying *mstnb* null alleles exhibited longer body lengths from 3 to 6 months post-fertilization [15]. The above results suggested that the knockout of *mstn* genes does not necessarily increase muscle or body size in fish.

The mammalian MSTN is encoded by a single gene, whereas some teleost fishes possess multiple orthologous or paralogous *mstn* genes. For instance, only a single *MSTN* gene was found in the medaka genome [32] and Japanese flounder [42], but two paralogous MSTN genes (*mstna* and *mstnb*) were identified in zebrafish [15,43,44], yellow catfish [10,11], rainbow trout [45], and grass carp [46]. Four types of *MSTN* genes (*mstn-1a*, *-1b*, and *mstn-2a*, *-2b*) in Atlantic salmon suggested at least two separate gene duplication events in this fish [47]. The studies in zebrafish, red sea bream, and yellow catfish suggested that *mstna* and *mstnb* might have evolved separately and have different expression patterns and functions [17,45,47] in adult tissues. According to the phenotypic changes in mstn knockout yellow catfish, we presume that one *mstn* mutant could increase muscle mass [12], whereas another *mstn* mutant affects body length.

A preliminary study of *mstn* functional inactivation in mammals investigated the differences in muscle mass. Some studies have pointed out that myostatin affects bone development and growth in mammals and is involved in bone metabolism. *mstn* increases muscle mass, leading to an enhancement of the strength of the bones to which they are attached, especially the joints which become thicker or larger to withstand greater forces [23,24]. Hamrick [48] pointed out that inhibitors of myostatin function may be associated with increased muscle mass and bone mineral density (BMD). Elkasrawy and Hamrick [49] discovered that myostatin-deficient mice had a higher trabecular area and trabecular bone mineral content. With longer and shortened mandibles in the vertical dimension, adult MSTN-KO mice have significantly different mandibular shapes than WT mice. Significantly shorter cranial vault lengths and maxillary lengths were generally found in adult MSTN-KO mice [50]. Blocking MSTN with a polyclonal antibody (MsAb) could increase BMD, bone volume over total volume (BV/TV), trabecular number and thickness, and decrease trabecular separation and the structure model index [51]. The combination of weight-bearing training and MsAb could augment bone formation to a greater degree than a single treatment in rats [52]. Kellum et al. [53] also found that the fracture callus size and callus bone volume increased in *mstn*-deficient mice. Zhang et al. [54] found pelvic tilt, teeth dislocation, and tongue enlargement in the *mstn* mutant rabbits. They posited that the increased muscle mass and strength play essential roles in bone morphology. It is worth noting that Qian et al. [55] found that 20% of MSTN-KO Meishan pigs had one extra thoracic vertebra and this phenomenon may be due to the inhibition of myostatin function.

The vertebral column is an essential organ in fish, which plays a crucial role in support, protection, and movement. It is generally believed that the variation in fish body length is related to the abnormal development of vertebrae; that is to say, the variation in vertebrae number or length causes length differences between individuals. M. Hattori et al. [31] found that vertebral deformities such as centrum defects and undersized centra in red sea bream could cause changes in body length. Lindsey [56] pointed out that vertebral number was correlated with maximum body length among related fish species. Variations in the number of vertebrae are prevalent within and among fish species. The vertebral counts are determined by geographic distribution [57], living environment [58], hormone levels [28], and expression levels of related genes [59], but some research indicates that most fish have a fixed number of vertebrae during early ontogeny [57,60]. In this study, *mstnb* knockout yellow catfish were found to have decreased centrum counts. This is consistent with the result that the HBL ratios of *mstnb* yellow catfish were higher than those of their wild-type littermates. The mutant and wild yellow catfish were siblings and cultured in the exact same conditions, thereby reducing the environmental and genetic differences. All of our findings imply that the shorter body length of the mutant yellow catfish was caused by variations in vertebral bones, particularly centrum counts.

The number of vertebrae in fish predetermines body shape, body length, and swimming type [56] and, therefore, it is also related to feeding and predator avoidance [57]. The increased vertebral numbers would increase flexibility, allowing the fish to have multiple bends in the body and facilitating the adaptation to highly structured habitats. In contrast, low vertebral numbers in fish would cause reduced swimming performance, increased prey vulnerability, and a food fight disadvantage [61,62]. The growth data (body weight, body length, etc.) of the *mstnb* mutant yellow catfish revealed a decreasing trend compared to the wild type, indicating reduced growth, which also appeared to be compatible with the correlation between the vertebral number and individual size in this study. Precaudal and caudal vertebra sections from the trunk and rump segments of mutant yellow catfish (*P. fulvidraco*) were compared in this study, and it was discovered that after *mstnb* knockout, the vertebrae length in both areas was reduced (Figure 5). Additionally, the mutant yellow catfish has a shorter intervertebral length than the wild type. The CL/CW ratio also shows that the BK yellow catfish’s trunk and rump centra were larger than those of the WT fish.

Intramembranous ossification and endochondral ossification are two methods of bone formation in vertebrates. Endochondral ossification, in which the bones in the spine first form cartilage and then ossify, is the primary process by which spinal bone is created. Vertebral spacing was reduced, and bone mineral density was elevated in mice after *mstn* knockout [63]. Vertebral spacing shrank, and bone mineral density rose in mice after *mstn* deletion. Adult myostatin-deficient mice had a smaller femoral head than the wild group, and juvenile myostatin-deficient mice had less cartilage in their proximal femoral joints. These findings imply that *mstn* influences bone development by encouraging cartilage mineralization. In the bone tissues of the trunk and tail, the number and density of osteocytes were more significant in *mstnb* knockout *P. fulvidraco* than in wild-type *P. fulvidraco*. This is consistent with the findings that knockout of the *mstn* gene in mice enhances bone strength and density and that the intervertebral distance is reduced in knockout mice.

The effects of *mstnb* gene mutation on the full length and body length were mainly found on 180 and 250 dpf in females and 180 dpf in males. However, no significant difference between *mstnb* mutants and WT fish was found in a later stage. The possible reasons for the stage-dependent functions of *mstnb* include: *P. fulvidraco* is a small fish, and the maximum individual length is only 20–25 cm under normal conditions. In the early growth period, the differences in the number of vertebrae, vertebral length, and inter-vertebral distances do not show up clearly in the case of shorter body lengths. The effects of vertebral variation on fish growth and feeding were discussed above, and our growth data are almost always consistent with this theory at all ages. In the present study, the head length/body length ratio of *mstnb* mutation was increased. This suggests that the growth rate of mutant individuals accelerates significantly during the later stages of growth. This inference is also consistent with the trend of weight gain in mutant individuals. According to the published articles [64,65], the expression of the *mstna* and *mstnb* genes is stage-specific. Therefore, the effects of the knockout on the skeleton also show stage-specific differences.

Previous *mstna* knockout studies revealed that *mstna* significantly increases muscle mass in yellow catfish but has no appreciable impact on body weight or length. Similar cases had been reported in *Pm-mstn* knockout red sea bream [17]: the mutant adult fish had lower weights than the wild-type individuals, and the body length decreased significantly. The CT analysis found that mutant individuals showed short centrum, intervertebral, and head lengths. All these changes combined resulted in shorter body lengths in the mutants. However, *mstnb* functional inactivation does not impact body length and weight [17]. Although the gene functions of *mstna* and *mstnb* may differ in different species, the overall function of the mstn genes remained the same, regulating muscle growth, influencing bone, and shortening body length. Mstn interference or knockout led to individual magnification in some zebrafish and killifish studies [16]. Although the skeletal condition was not examined, it was determined that the skeletal changes were unavoidable.

Taken together, the *mstn* mutation caused mutations in the number and length of vertebrae as well as the intervertebral distance in *Pelteobagrus fulvidraco*, resulting in a shorter body length in the mutant *Pelteobagrus fulvidraco*.

## 5. Conclusions

In conclusion, the *mstnb* mutant in *Pelteobagrus fulvidraco* results in a smaller length than the wild-type and a lower body weight than the wild-type at the same stage. Our results demonstrated that when using genome editing technology to breed new lines, the effects of knockout need to be analyzed comprehensively and may have some unexpected effects due to insufficient study of the function of certain genes.

## Figures and Tables

**Table 1:** The morphological data in *mstnb* null yellow catfish and their wild-type siblings at 90, 180, 250 and 360 dpf.

		Male						Female					
		BK Group	WT Group	MeanDiff (95%CI)	t	df	*p*	BK Group	WT Group	MeanDiff (95%CI)	t	df	*p*
90	body weight (g)	10.70 ± 4.08	11.60 ± 3.58	−0.90 (−2.93, 1.13)	−0.886	77.000	0.379	8.05 ± 1.96	8.85 ± 1.88	−0.80 (−1.65, 0.06)	−1.843	77.000	0.069
	full length (cm)	10.41 ± 1.30	10.99 ± 1.27	−0.58 (−1.27, 0.10)	−1.708	77.000	0.093	9.57 ± 0.77	9.88 ± 0.73	−0.29 (−0.58, 0.00)	−2.023	77.000	0.047
	body length (cm)	8.75 ± 1.11	9.28 ± 1.12	−0.53 (−1.13, 0.06)	−1.796	77.000	0.078	8.02 ± 0.658	8.32 ± 0.63	−0.31 (−0.64, 0.03)	−1.818	77.000	0.073
	body height (mm)	20.33 ± 2.73	20.71 ± 2.23	−0.38 (−1.70, 0.93)	−0.584	77.000	0.561	18.39 ± 1.75	18.94 ± 1.46	−0.55 (−1.27, 0.17)	−1.516	77.000	0.134
	body width (mm)	14.19 ± 1.89	14.40 ± 1.80	−0.21 (−1.19, 0.78)	−0.423	77.000	0.674	12.82 ± 1.41	13.38 ± 1.22	−0.56 (−1.15, 0.03)	−1.896	77.000	0.062
180	body weight (g)	32.36 ± 9.17	42.40 ± 9.51	−10.04 (−15.33, −4.75)	−3.799	44.000	0.000	15.16 ± 3.14	21.76 ± 5.71	−6.60 (−9.30, −3.89)	−4.915	44	0.000
	full length (cm)	14.75 ± 1.48	16.34 ± 1.26	−1.59 (−2.34, −0.85)	−4.279	44.000	0.001	11.55 ± 0.76	12.87 ± 1.09	−1.32 (−1.87, −0.76)	−4.795	44	0.000
	body length (cm)	12.44 ± 1.36	13.87 ± 1.11	−1.42 (−2.09, −0.76)	−4.795	44.000	0.000	9.69 ± 0.67	10.92 ± 0.96	−1.23 (−1.72, −0.74)	−5.043	44	0.000
	body height (mm)	28.38 ± 2.49	31.00 ± 2.56	−2.62 (−4.06, −1.19)	−3.813	35.757	0.001	21.71 ± 1.93	25.32 ± 2.72	−3.61 (−5.00, −2.22)	−5.2284	44	0.000
	body width (mm)	18.83 ± 1.91	20.36 ± 1.81	−1.52 (−2.56, −0.49)	−5.228	44.000	0.000	14.58 ± 1.43	16.88 ± 1.87	−2.30 (−3.28, −1.31)	−4.7056	44	0.000
	head length (cm)	3.26 ± 0.34	3.51 ± 0.26	−0.24 (−0.41, −0.08)	−4.706	44.000	0.001	2.54 ± 0.15	2.76 ± 0.23	−0.22 (−0.34, −0.10)	−3.81334	35.75654	0.001
250	body weight (g)	57.19 ± 23.55	58.54 ± 18.89	−1.35 (−17.23, 14.53)	−0.174	28.000	0.863	23.46 ± 7.58	26.98 ± 6.23	−6.60 (−9.30, −3.89)	−4.915	36	0.127
	full length (cm)	17.46 ± 2.45	18.04 ± 2.06	−0.58 (−2.27, 1.10)	−0.710	28.000	0.483	12.81 ± 1.48	13.59 ± 0.94	−1.32 (−1.87, −0.76)	−4.79503	31.065	0.040
	body length (cm)	14.87 ± 2.17	15.51 ± 1.83	−0.64 (−2.14, 0.86)	−0.867	28.000	0.388	10.83 ± 1.24	11.56 ± 0.81	−1.23 (−1.72, −0.74)	−5.04327	30.528	0.127
	body height (mm)	33.87 ± 4.61	34.98 ± 4.25	−1.11 (−4.45, 2.22)	−0.688	28.000	0.497	26.09 ± 3.44	28.29 ± 3.15	−3.61 (−5.00, −2.22)	−5.2284	36	0.060
	body width (mm)	23.95 ± 4.31	22.80 ± 3.09	1.16 (−1.62, 3.94)	0.835	23.244	0.412	18.17 ± 2.82	18.81 ± 1.95	−2.30 (−3.28, −1.31)	−4.7056	36	0.061
	head length (cm)	3.86 ± 0.54	3.87 ± 0.43	−0.01 (−0.37, 0.35)	−0.064	28.000	0.950	2.86 ± 0.33	2.88 ± 0.20	−0.22 (−0.34, −0.10)	−3.81334	36	0.001
360	body weight (g)	94.36 ± 41.07	104.98 ± 41.95	−10.62 (−32.39, 11.15)	−0.977	57.000	0.333	36.04 ± 10.32	37.34 ± 12.00	−1.30 (−7.35, 4.75)	−0.431	53.000	0.668
	full length (cm)	20.49 ± 3.32	21.38 ± 3.38	−0.90 (−2.66, 0.86)	−1.021	57.000	0.311	14.64 ± 1.48	14.84 ± 1.42	−0.21 (−0.99, 0.58)	−0.523	53.000	0.603
	body length (cm)	17.22 ± 2.89	17.92 ± 2.93	−0.69 (−2.22, 0.83)	−0.911	57.000	0.366	12.18 ± 1.32	12.42 ± 1.28	−0.24 (−0.95, 0.47)	−0.682	53.000	0.498
	body height (mm)	40.27 ± 6.63	42.20 ± 7.49	−1.92 (−5.67, 1.83)	−1.044	57.000	0.301	30.54 ± 3.36	30.90 ± 3.69	−0.36 (−2.27, 1.55)	−0.378	53.000	0.707
	body width (mm)	28.72 ± 4.27	30.07 ± 4.75	−1.35 (−3.71, 1.00)	−1.149	57.000	0.255	22.10 ± 2.48	22.25 ± 2.63	−0.15 (−1.53, 1.23)	−0.222	53.000	0.825

*p*-value <0.01 was considered significant, as we had to correct our analysis for multiple testing (*p*-value of 0.017 was calculated as: 0.05 divided by 5).

## Data Availability

The data presented in this study are available in the article. Further information is available upon request from the corresponding author.
